# Numerical Analysis of Stress and Temperature Fields in a Composite Stratum Based on a New Method of Shield Construction for Safety and Environmental Protection

**DOI:** 10.3390/ijerph17020530

**Published:** 2020-01-14

**Authors:** Wei Dai, Yimin Xia, Bo Ning, Mei Yang

**Affiliations:** 1 College of Mechanical and Electrical Engineering, Central South University, Changsha 410083, China; deway2008@csu.edu.cn (W.D.); 173701016@csu.edu.cn (B.N.); yangmeicsu@163.com (M.Y.); 2 China Railway Construction Heavy Industry Co., LTD, Changsha 410100, China

**Keywords:** tunnel construction, frozen cutter head, composite stratum, analysis of temperature field

## Abstract

Safety and environmental protection are key issues in shield construction. Due to wear, the cutter of a shield machine must be changed after a period of excavation. In order to realize the tool change operation of a shield machine at atmospheric pressure, a method of cutter head freezing of the shield machine is described in this paper. The finite element simulation method is used to analyze the construction of a shield machine with a frozen cutter head in a composite stratum. For a composite stratum with uneven hard and soft layers in a ratio of 1:1, the stress and temperature fields are analyzed, and the stress change around the hob is recorded. Through numerical simulation, the change of the temperature field around the shield machine is determined in real time. As time goes on, the temperature around the shield machine decreases, and the frozen range expands. When the temperature field in a specific point reaches a critical value, the temperature at that point will remain constant, and the stress field around the cutter head will also tend to become stable. The isothermal region of the soil presents an annular distribution, and the final temperature tends to be stable and gradually increase as the distance from the frozen cutter head increases. The final temperature of the monitoring area reaches a stable value corresponding to −26.5 °C, the axial depth of the frozen wall is more than 2.5 m, the minimum frozen radius is 3.2 m, the stress distribution around the cutter head is unbalanced, the maximum stress is measured in the hard rock layer, and the stress around the cutter head at the hob level indicates that tool change is necessary. Compared with the traditional method, the construction method of a frozen cutter head is more effective and more environmentally friendly. Further research will allow a broad application of this method in shield excavation in a composite stratum.

## 1. Introduction

With the increasing traffic congestion, the construction of underground space has become a hot topic. Shield construction is a widely used method to build tunnels. Scholars have begun to pay attention to safety and environmental protection in subway and tunnel construction [1,2]. In the process of shield tunneling, the cutter head body and the cutters installed on the cutter head face are in direct contact with the excavated soil. Because of mutual friction and collision, the cutter head body is prone to cracking causing abnormal wear, and other failures. During installation, the cutter head body is prone to eccentric wear, sealing failure, edge collapse, and other types of failure [3,4,5]. Cutter wear is the main failure phenomenon occurring in the construction of a shield machine. When the corresponding failure phenomenon occurs during field construction, it is necessary to properly manage the failed tool and cutter head [6,7,8]. During excavation, when the shield construction passes through a hard–soft heterogeneous stratum, the high applied pressure will be released on the cutter head. This could seriously worn and damage the hob and reduce the strength and rigidity of the cutter head. Therefore, in such a situation, it is necessary to often modify the working technique and adjust the excavation parameters, frequently changing the tools utilized.

If not handled properly, this type of situation can easily deforme the surface of the ground. Many scholars have carried out research work on this. For example, Cao et al. [9] put forward a calculation method of strata heave caused by shield construction under a double-layer elastic system. The calculation method they used can simultaneously consider the influence of six construction factors on strata heave, such as the unbalanced force at the excavation face and the friction between the shield shell and the soil mass. Based on the construction of subway tunnels in soft soil, the basic law of vertical deformation caused by the construction of a rectangular shield tunnel was analyzed by Si et al. [10]. Gonzalez and Sagaseta [11] obtained the specific values of surface loss, tunnel elliptization, and soil volume deformation by fitting the measured displacement in a Madrid metro tunnel construction project and studied how each parameter was affected by changes in tunnel geometry size and soil type.

The main maintenance methods include normal pressure maintenance and pressure entry maintenance into the warehouse. The pressure maintenance methods have a wide range of applications but they require high technical and physical ability of the construction personnel; also, the safety risk is high, and engineering accidents are frequent. The working conditions when operating at atmospheric pressure operation are better than those of operating under pressure; the former method requires lower technical skills but is more demanding regarding the conditions of the construction stratum. In order to perform normal-pressure operations, the existing technology often adopts the artificial ground-freezing method, which consists of laying multiple cooling pipes which will freeze the surrounding soil, creating a waterproof seal reinforcing the stratum [12,13,14]. However, due to improper operation, cooling pipe frost crack, and other reasons, the waterproof seal may break, and the whole stratum may collapse.

Therefore, a reliable technology for normal-pressure cutter change operation of a shield machine is urgently needed. Besides, time is also one of the issues that need to be paid attention to in shield construction. The grouting reinforcement method is often used in shield construction. Considering the setting time of cement grouting and the time required for tool change, the downtime is often more than one month [15,16,17]. It is important to effectively reduce the operation time and the construction cost of a shield machine (of the cutter head and due to cutter wear), thus reducing the risk of the cutter change operation for the construction personnel [18].

This paper proposes a shield machine’s cutter head freezing method for the construction of a shield under a composite stratum. A cooling pipeline system is laid on the back of the cutter head and inside the shield body. In this way, even if the frozen cooling pipes become cracked, the frozen soil will not melt and collapse. Compared with the traditional freezing method, the cutter head freezing method of a shield machine for tunnel construction, is safer, more reliable, energy-saving, and environmentally friendly. Through numerical simulation analysis of the temperature field and stress field distribution around the cutter head of the shield machine, it provides a new way to solve the problem of cutter change in a composite stratum.

## 2. Transformation of the Freezing Cutter Head 

**Figure 1:**
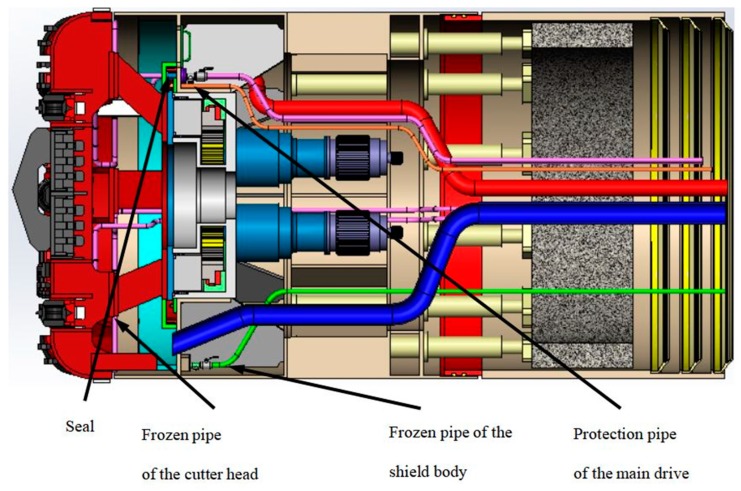
Transformation of the cutterhead.

**Figure 2:**
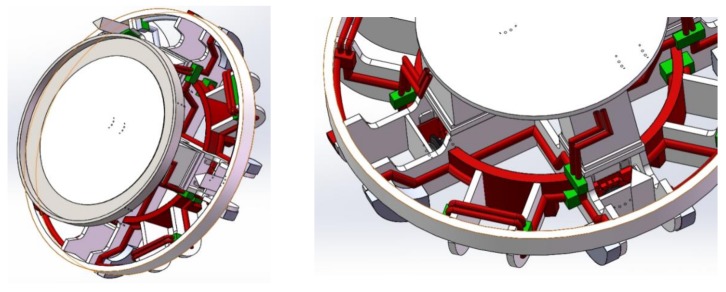
Distribution of the frozen pipeline in the cutter head.

When the cutter of a shield machine is worn or at risk of damage, it is necessary to open the machine for inspection and possibly change the cutting tools. We propose to transform the cutter head of a φ 4350 shield machine by adding a freezing pipeline and equipment, so that the cutter head of the shield machine acquires the function of freezing the ground. By freezing and reinforcing of the soil layer outside the soil chamber, it is then possible to open and change the cutting tools under normal pressure. A freezing test was carried out after completing the cutter head transformation and before the construction of the well. This verified that opening and changing of the cutter during shield construction was possible. The transformation consisted in reconstructing the cutter head and front shield of the slurry-balanced shield machine, adding the cutter head freezing pipeline, the shield body freezing pipeline, and the main drive protection pipeline, connecting the pipe sealing chamber, etc., as shown in Figure 1 and Figure 2.

## 3. Freezing Control Model

The refrigeration cycle system consisted of soil, cutter head, freezing tube set, liquid refrigerant, and refrigeration unit. In the process of soil freezing, energy migrates and changes along a certain route. When the internal moisture reaches the critical freezing temperature, the soil begins to freeze, and a cold front in the soil begins to form. Then, energy enters the refrigerant by means of heat conduction through the cutter head, and the temperature increases accordingly. Finally, the heat is taken away and released through the cooling circulation system.

### 3.1. The Law of Energy Conservation

It is assumed that the whole freezing process of soil is completed in an ideal state without energy loss, so the first law of thermodynamics (the law of energy conservation) is always valid throughout the cooling cycle [19], according to Equation (1):

(1) $$-\Delta E_{s}+\psi_{q}=+\Delta E_{l}$$

where $\Delta E_{s}$ denotes the internal heat loss per unit volume of soil after freezing in a given time, $\psi_{q}$ denotes the icing latent heat per unit volume of soil in a given time, $\Delta E_{l}$ denotes the total heat energy removed from the soil by a cryogenic refrigerant in a given time.

### 3.2. Mathematical Model of the Freezing Temperature Field

It is assumed that land is ideally homogeneous and continuous. If vertical heat transfer is not considered, the freezing temperature field can be simplified to an axisymmetric plane problem [20]:

(2) $$\frac{\partial t_{n}}{\partial \tau }=\alpha_{n}\left(\frac{\partial^{2}t_{n}}{\partial r^{2}}+\frac{1}{r}\frac{\partial t_{n}}{\partial r}\right)$$

where $t_{n}$ denotes the temperature distribution, *n* denotes the soil state, *n* = 1 denotes the melting soil, *n* = 2 denotes the frozen soil, τ denotes the freezing time, *r* denotes cylindrical coordinates with the center of the freezing pipe as the origin, $\alpha_{n}$ denotes the thermal conductivity coefficient.

Boundary condition: before freezing begins, the soil has a uniform temperature *t*_0_; at infinite distance, the temperature field is not affected by freezing. So:

(3) $$t\left(\infty ,\tau \right)=t_{0}$$

(first kind of boundary conditions)

On the frontal surface of the freezing wall (the zero-degree surface), the freezing temperature is always the following

(4) $$t\left(\zeta_{N},\tau \right)=t_{d}$$

(first kind of boundary conditions)

On both sides of the frontal surface of the freezing wall, we have:

(5) $$\lambda_{2}\left|{\frac{\partial t_{2}}{\partial r}}_{r=\xi_{N}}\right.-\lambda_{1}\left|{\frac{\partial t_{1}}{\partial r}}_{r=\xi_{N}}\right.=\delta \frac{d\zeta_{N}}{d\tau }$$

(first kind of boundary conditions)

On the arranged freezing pipes, the heat exchange conditions inside and outside the freezing pipes are as follows.

(6) $$\lambda \left|{\frac{dt_{n}}{dr}}_{r=R_{0}}\right.=\alpha \left(t_{w}-t_{c}\right)$$

(third kind of boundary conditions)

(7) $$t\left(R_{0},\tau \right)=t_{0}$$

(first kind of boundary conditions), where $t_{0}$ denotes the initial temperature of the soil, $t_{d}$ denotes the freezing temperature of the soil, $t_{w}$ denotes the temperature on the wall of the freezing pipes, $t_{c}$ denotes the brine temperature, $\lambda_{1},\lambda_{2}$ denote the thermal conductivity of thawed and frozen soils, respectively, $R_{0}$ denotes the radius of the freezing pipes, R denotes the freezing influence radius, $\zeta_{N}$ denotes the coordinates of the frontal surface of the freezing wall in the N region, so that, when *N* = 1, $0\leq \zeta_{1}\leq R_{0}$, when *N* = 2, $R_{0}\leq \zeta_{2}\leq R$, σ denotes the unit volume of latent heat of the frozen soil.

## 4. Numerical Simulation

**Figure 3:**
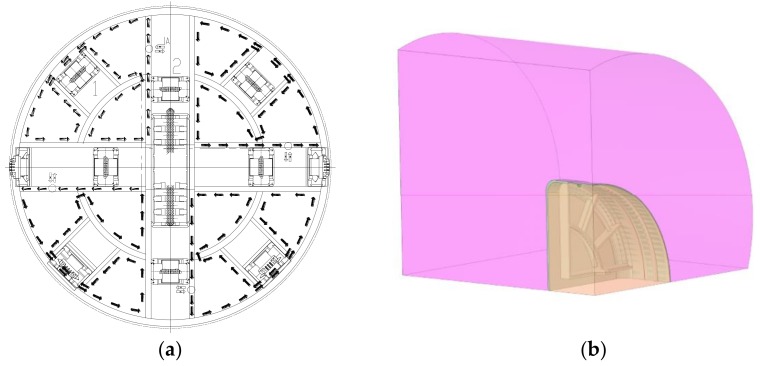
Geometric model of the frozen soil system of the cutter head. (**a**): Geometric model of the cutter head; (**b**): Frozen soil system of the cutter head

According to the drawings provided by China railway construction heavy industry Co., Ltd., a full-section model of a shield cutter head was established considering its symmetry, the visibility of the temperature field change, and the amount of calculation required. In order to ensure the feasibility of analysis and calculation, some chamfers and small gaps of the cutter head were omitted in the solid modeling process. The soil mass was represented by a cylinder with a diameter of 7 m and a depth of 5 m. The internal clearance of the cutter head was filled with soil. The geometry of the cutter head frozen soil system is shown in Figure 3.

Solid70 is a three-dimensional solid element, and Solid70 is an eight-node hexahedral element, which has isotropic and three-dimensional heat transfer capability. The element has eight nodes and only one temperature degree of freedom on each node. It can be used for three-dimensional static or transient thermal analysis and can achieve uniform heat flow transfer. When the model is meshed, the accuracy and time of calculation should be considered comprehensively. The grid density should be increased in the area near a frozen pipe and in the region where the soil will freeze. In the area far from the frozen pipes, the grid density should be reduced, as the temperature gradient is small.

The cutter head was made of Q345 steel, with a heat conductivity of 70 w/m·°C a density of 7800 kg/m^3^, and a specific heat of 448kj/kg·° C. The thermo-physical parameters and mechanical parameters of soft rock and hard rock were obtained through laboratory tests, as shown in Table 1 and Table 2 [21]. The thermodynamic parameters of the two soils were basically the same, but the mechanical parameters differed greatly.

According to the field test data, the boundary conditions were set as follows:

(1) the initial ground temperature was determined according to the data obtained for the freezing project of a shallow tunnel, and the initial soil temperature before freezing was 18 °C;
(2) the symmetrical surface of the geometric model was the adiabatic boundary, the cylindrical surface outside the soil layer was the constant temperature boundary, the interior of the freezing pipe was the convection heat exchange interface, the convection coefficient was set to 110 W/m^2^, and the coolant temperature was set to −30 °C.

**Figure 4:**
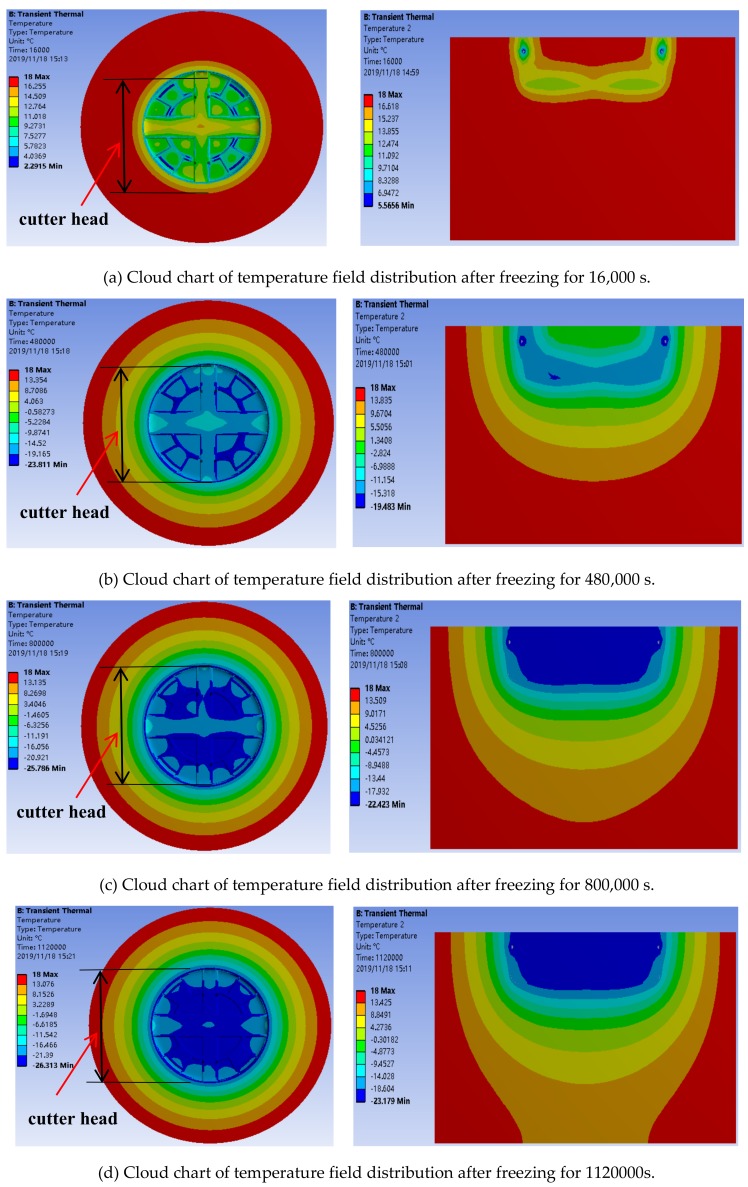

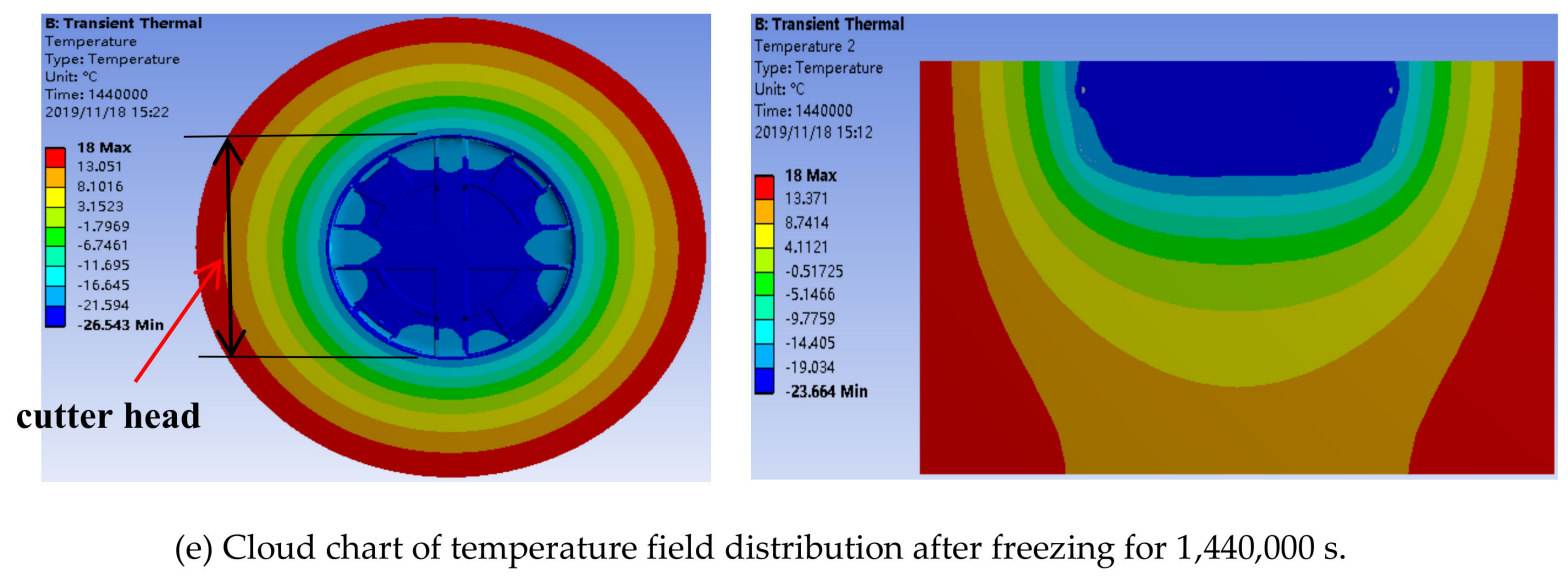
Temperature field contours of the frozen soil around the cutter head.

The cylindrical soil was considered to be composed of layers, i.e., an upper soft layer and a lower hard layer in a ration of 1:1 for analysis and calculation, the other parameters were assumed to remain unchanged. The cloud chart of the numerical analysis under the condition of uneven, hard and soft (1:1) soil, is shown in Figure 4.

According to the established finite element calculation model and the determined typical parameters, the temperature change in the frozen soil layer and the corresponding cloud chart of were determined. Figure 4 shows the development of the temperature field around the freezing soil in 15 days.

**Figure 5:**
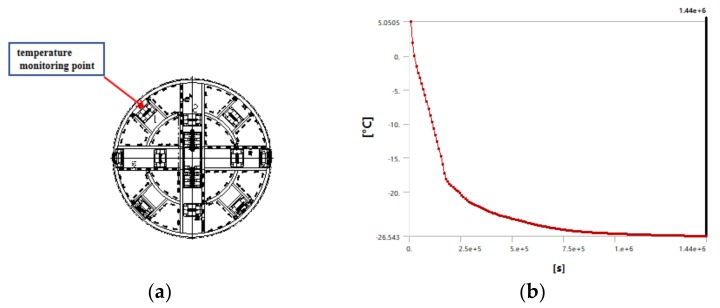
Temperature monitoring point and time-dependent curve of minimum soil temperature. (**a**): Temperature monitoring point; (**b**): time-dependent curve of minimum soil temperature.

Through numerical simulation, we described the change of the temperature field around the shield machine in real time. As time went on, the temperature around the shield machine kept decreasing, and the freezing range kept expanding outwards. After a certain point in the temperature field reached a critical temperature value, the temperature at that point remained constant. Under the assumption of a uniform soil, because the parameters of temperature transfer in the two kinds of soil are similar, the isothermal region is distributed in a ring. As the distance from the freezing cutter head increases, the temperature tends to become stable. The curve representing the change of the minimum temperature with time in the monitoring area is shown in Figure 5; as shown, the temperature reached a final stable value of −26.5 °C. After the cutter head was frozen for 360 h, the axial depth of the frozen wall was more than 2.5 m, and the minimum freezing radius was 3.2 m.

**Figure 6:**
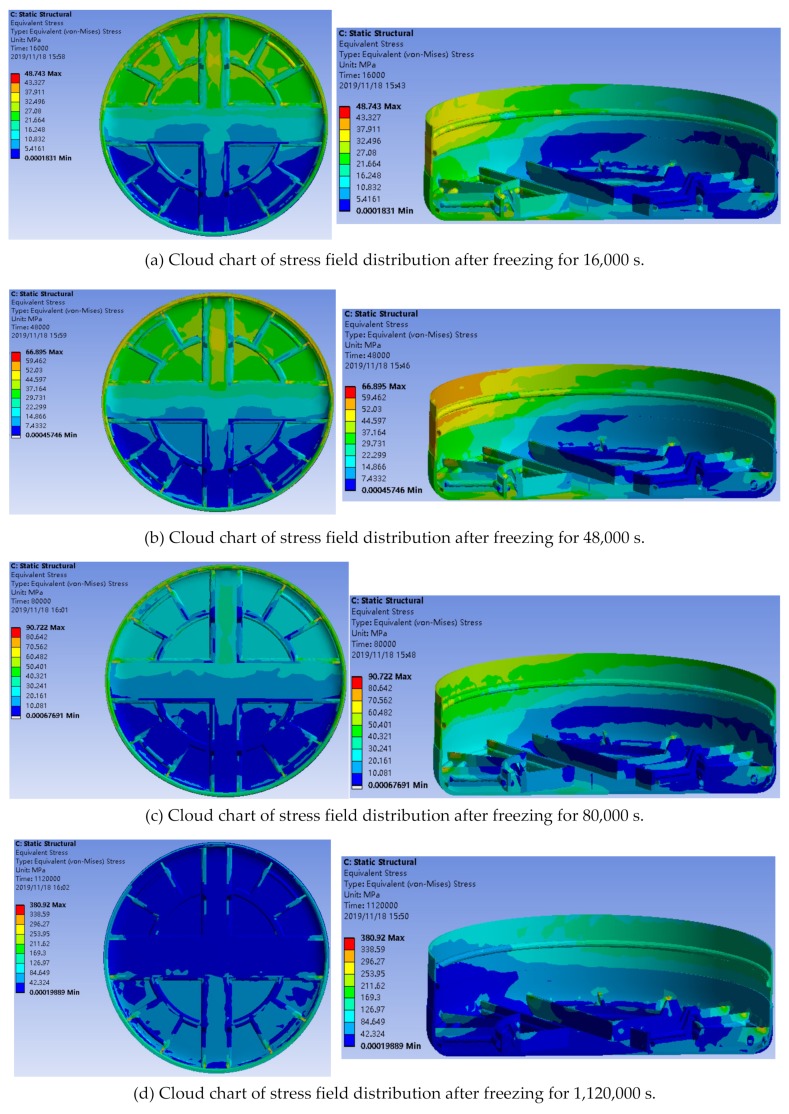

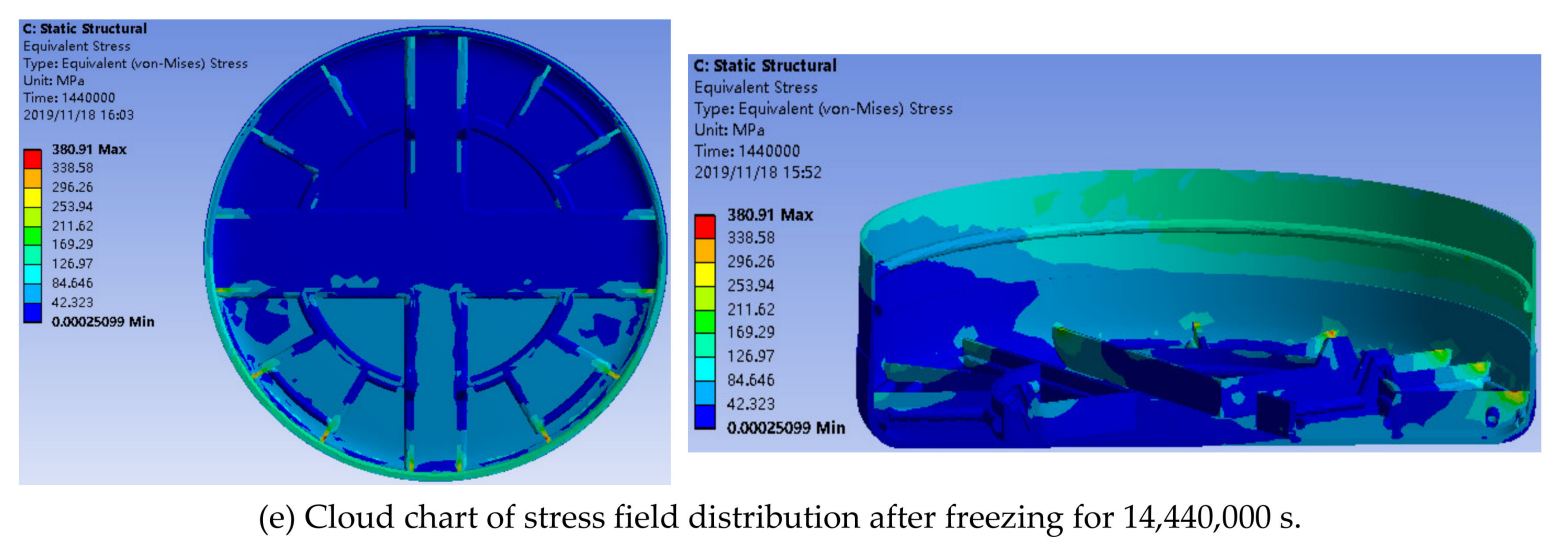
Stress field contours of the cutter head.

By analyzing the stress field change around the tool during the freezing process and checking the thickness and strength of the frozen wall after 360 h of continuous freezing, the stress distribution around the whole cutter head was monitored in real time, as shown in Figure 6. It can be seen from Figure 6 that the cutter head was in an unbalanced stress state, and the stress was relatively concentrated in the area of hard rock.

**Figure 7:**
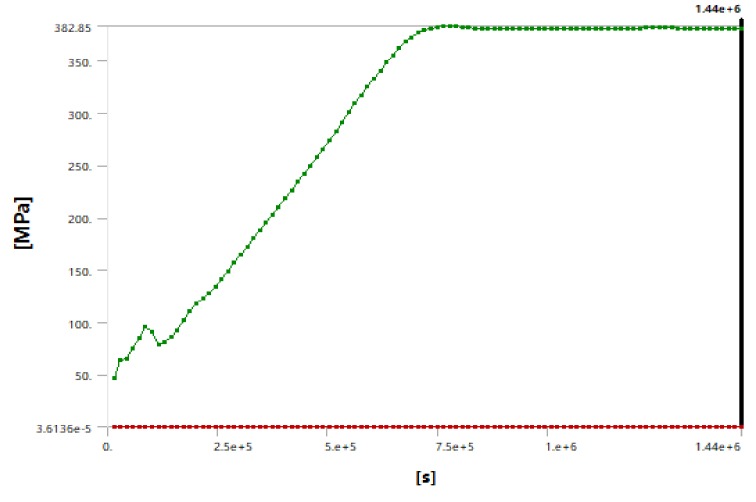
Stress–time curve for the cutter head.

**Figure 8:**
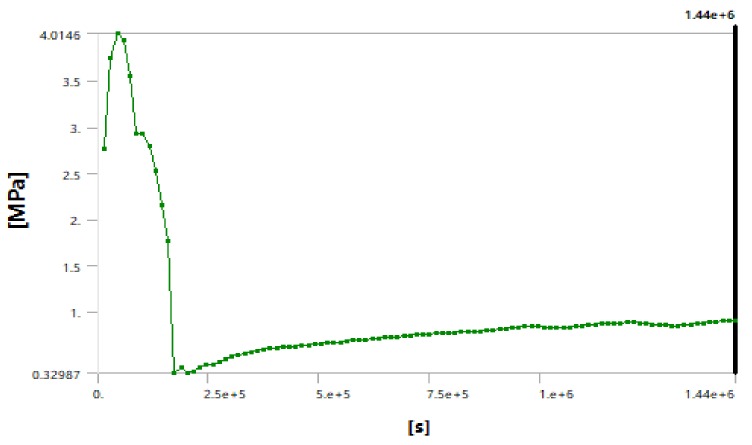
Stress–time curve for the surface between the soft rock layer and the cutter head

**Figure 9:**
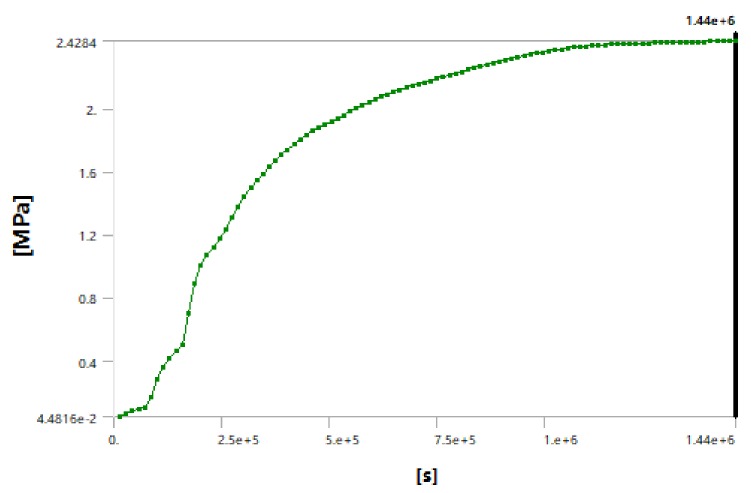
Stress–time curve for the surface between the hard rock layer and the cutter head.

Through numerical simulation, we described the change of the stress field around the cutter head of the shield machine in real time. The maximum stress of the cutter head increased with the decrease of the surrounding temperature over time, as shown in Figure 7. As shown in Figure 8 and Figure 9, after the temperature field was stabilized the stress of the soil layer near the cutter head remained constant after reaching a critical state. By monitoring the stress near the hob, we found that the stress in the soil layer near cutter head was 2.4 Mpa (hard rock) and 0.9 Mpa (soft rock), which indicated that the cutter can be changed in this case.

## 5. Conclusions

For the sake of environmental protection and safety, a new method of cutter head freezing in shield construction was presented, and a numerical simulation analysis of the temperature field and stress field around the cutter head in a composite soil was carried out. Our conclusions are as follows:

(1) Through numerical simulation, the change of the temperature field around the shield machine could be determined in real time. As time went on, the temperature around the cutter head decreased, and the freezing range expanded outwards. After a certain point in the temperature field reached a critical value, the temperature at that point remained constant, and the stress field of the cutter head also tended to stabilize.
(2) In the case of uneven (1:1) soil, the isothermal region of the two types of soils presented a ring distribution due to the similar temperature transfer in the two types of soil.
(3) With the distance from the freezing cutter head increasing, the final temperature of the soil gradually increased. The final temperature of the monitoring area was stable at −26.5 °C, the axial depth of the frozen wall was more than 5 m, and the minimum freezing radius was 3.2 m.
(4) In the soft and hard uneven strata (1:1), the stress distribution around the cutter head was i unbalanced, the maximum stress (2.4 Mpa) was always measured in the hard soil layer, and the high stress around the cutter head at the hob position indicated that the cutter can be changed in this case.
(5) Compared with the traditional method, the method of cutter head freezing of a shield machine here presented can better meet the safety and environmental protection requirements of underground construction.

## Figures and Tables

**Table 1:** Thermal physical and mechanical parameters of the soil.

Temperature (°C)	Coefficient of Thermal Expansion	Thermal Conductivity(W/m·°C)	Specific Heat (J/Kg·°C)	Density (kg/m^3)^	Modulus of Elasticity (Pa)	Poisson’s Ratio	Tensile Strength (kPa)
−30	−9.6 × 10^−5^	6.3	810	1640	1.43 × 10^8^	0.28	2.35 × 10^4^
−28	−9.6 × 10^−5^	6.3	810	1640	1.34 × 10^8^	0.29	2.25 × 10^4^
−26	−9.5 × 10^−5^	6.3	810	1640	1.25 × 10^8^	0.29	2.15 × 10^4^
−24	−9.4 × 10^−5^	6.3	810	1640	1.1 × 10^8^	0.29	2.08 × 10^4^
−22	−9.4 × 10^−5^	6.3	810	1640	1.0 × 10^8^	0.29	1.96 × 10^4^
−20	−9.3 × 10^−5^	6.3	810	1640	9.8 × 10^7^	0.3	1.88 × 10^4^
−18	−9.3 × 10^−5^	6.3	810	1640	9.0 × 10^7^	0.3	1.80 × 10^4^
−16	−9.2 × 10^−5^	6.3	810	1640	8.2 × 10^7^	0.3	1.74 × 10^4^
−14	−9.2 × 10^−5^	6.3	810	1640	7.3 × 10^7^	0.3	1.65 × 10^4^
−12	−9.1 × 10^−5^	6.3	810	1640	6.3 × 10^7^	0.3	1.55 × 10^4^
−10	−9 × 10^−5^	6.3	810	1640	5.4 × 10^7^	0.3	1.45 × 10^4^
−8	−8.8 × 10^−5^	6.3	810	1640	4.3 × 10^7^	0.31	1.35 × 10^4^
−6	−8.5 × 10^−5^	6.3	810	1640	3.2 × 10^7^	0.31	1.25 × 10^4^
−4	−8.5 × 10^−5^	6.3	810	1640	2.8 × 10^7^	0.34	1.00 × 10^4^
−2	−8.4 × 10^−5^	6.3	810	1640	2.0 × 10^7^	0.37	4.80 × 10^3^
−1	−6.6 × 10^−5^	5.25	34000	1710	1.5 × 10^7^	0.39	3.50 × 10^3^
0	0	4.2	880	1780	1.0 × 10^7^	0.41	2.00 × 10^3^
100	0	4.2	880	1780	1.0 × 10^7^	0.41	2.00 × 10^3^

**Table 2:** Thermal physical and mechanical parameters of the rock.

Temperature (°C)	Coefficient of Thermal Expansion	Thermal Conductivity (W/m·°C)	Specific Heat (J/Kg·°C)	Density (kg/m^3^)	Modulus of Elasticity (Pa)	Poisson’s Ratio	Tensile Strength (kPa)
−30	−5.5 × 10^−5^	6.3	810	1723	7.15 × 10^8^	0.32	2.35 × 10^4^
−28	−5.4 × 10^−5^	6.3	810	1723	6.7 × 10^8^	0.33	2.25 × 10^4^
−26	−5.4 × 10^−5^	6.3	810	1723	6.25 × 10^8^	0.33	2.15 × 10^4^
−24	−5.3 × 10^−5^	6.3	810	1723	5.5 × 10^8^	0.33	2.08 × 10^4^
−22	−5.2 × 10^−5^	6.3	810	1723	5.0 × 10^8^	0.33	1.96 × 10^4^
−20	−5.2 × 10^−5^	6.3	810	1723	4.9 × 10^8^	0.34	1.88 × 10^4^
−18	−5.1 × 10^−5^	6.3	810	1723	4.5 × 10^8^	0.34	1.80 × 10^4^
−16	−5 × 10^−5^	6.3	810	1723	4.1 × 10^8^	0.34	1.74 × 10^4^
−14	−4.8 × 10^−5^	6.3	810	1723	3.65 × 10^8^	0.34	1.65 × 10^4^
−12	−4.8 × 10^−5^	6.3	810	1723	3.15 × 10^8^	0.34	1.55 × 10^4^
−10	−4.7 × 10^−5^	6.3	810	1723	2.7 × 10^8^	0.34	1.45 × 10^4^
−8	−4.5 × 10^−5^	6.3	810	1723	2.15 × 10^8^	0.35	1.35 × 10^4^
−6	−4.4 × 10^−5^	6.3	810	1723	1.6 × 10^8^	0.35	1.25 × 10^4^
−4	−4.3 × 10^−5^	6.3	810	1723	1.4 × 10^8^	0.38	1.00 × 10^4^
−2	−4.1 × 10^−5^	6.3	810	1723	1.0 × 10^8^	0.41	4.80 × 10^3^
−1	−3.5 × 10^−5^	5.25	34000	1796	7.5 × 10^7^	0.43	3.50 × 10^3^
0	−2.1 × 10^−5^	4.2	880	1870	5.0 × 10^7^	0.45	2.00 × 10^3^
100	0	4.2	880	1870	5.0 × 10^7^	0.45	2.00 × 10^3^
